# Compound sulfamethoxazole for pediatric pertussis: a retrospective cohort study in a region with high macrolide resistance

**DOI:** 10.3389/fped.2025.1622467

**Published:** 2025-12-09

**Authors:** Lin Li, Taohong Xie, Liu Xiao, Ying Zhong, Mingying Wang, Yaping Su

**Affiliations:** Department of Pediatrics of Ziyang People’s Hospital, Ziyang, Sichuan, China

**Keywords:** whooping cough, child, therapeutics, compound sulfamethoxazole, macrolide resistance

## Abstract

**Background:**

In recent years, the incidence of pertussis has been increasing globally. The high prevalence of macrolide-resistant strains has led to a significant rise in both pertussis cases and associated mortality.

**Objective:**

This retrospective study aimed to evaluate the clinical effectiveness and safety of compound sulfamethoxazole (SMZco) vs. macrolides in pediatric pertussis treatment under real-world clinical conditions, providing evidence for its clinical application.

**Methods:**

Patients were divided into macrolide and SMZco groups based on treatment regimens. Comparative analyses included overall treatment effectiveness, duration of nocturnal coughing, paroxysmal coughing, post-tussive vomiting, hospitalization length, and adverse drug reactions.

**Results:**

The macrolide group (*n* = 23) showed 3 markedly effective, 8 effective, and 12 ineffective cases (total effectiveness rate 47.8%). The SMZco group (*n* = 79) demonstrated 26 markedly effective, 38 effective, and 15 ineffective cases (total effectiveness rate 81.0%), with significantly superior effectiveness vs. macrolides (*P* < 0.05). The SMZco group exhibited statistically significant reductions in hospitalization duration (*P* < 0.05), paroxysmal coughing (*P* < 0.05), post-tussive vomiting (*P* < 0.05), and nocturnal coughing (*P* < 0.05). Gastrointestinal adverse events occurred in 8 macrolide-treated patients vs. 6 SMZco-treated patients (*P* < 0.05). Rash was observed in 12 SMZco-treated cases but none in the macrolide group (*P* < 0.05).

**Conclusion:**

SMZco significantly improves treatment effectiveness, shortens symptom duration and hospitalization, and reduces economic burden in pediatric pertussis. These findings position SMZco as an effective and safe alternative, particularly in regions with high macrolide resistance.

## 1 Introduction

Pertussis, an acute respiratory infectious disease caused by Bordetella pertussis, is characterized by paroxysmal spasmodic coughing often accompanied by inspiratory whooping sounds. Severe cases may present with facial flushing, apnea, cyanosis, and potentially fatal asphyxia, posing significant threats to young children (1). Since the introduction of diphtheria-tetanus-pertussis (DTP) vaccine into the World Health Organization's Expanded Program on Immunization in 1974, global pertussis incidence has markedly declined. In China, following the nationwide immunization program initiated in 1978, reported cases decreased from 100 to 200 per 100,000 population in the 1960s–1970s to less than 1 per 100,000 by the late 1990s, stabilizing below 0.2 per 100,000 during 2006–2010 (2).

Macrolides (e.g., azithromycin, erythromycin) have long served as the cornerstone of pertussis treatment, recommended by global guidelines for transmission control and symptom duration reduction. However, emerging evidence suggests a rising prevalence of macrolide-resistant genotypes among circulating Bordetella pertussis strains (3, 4). The high prevalence of macrolide-resistant strains has progressively diminished their effectiveness, leading to prolonged illness, increased complications, and elevated healthcare costs (5). China faces particularly severe challenges, with surveillance data indicating macrolide resistance rates of 70%–100% among clinical *B. pertussis* isolates (5, 6). In China, a marked upward trend in reported pertussis cases was observed between 2016 and 2019, with case numbers rising from 5,584 in 2016 to over 30,000 by 2019. The emergence of COVID-19 at the end of 2019 impacted the epidemiology of pertussis. From January to August 2019, pertussis incidence gradually increased; however, following the implementation of pandemic containment measures, case numbers began to decline. A sharp decrease to 4,475 cases was recorded in 2020. With subsequent adjustments to COVID-19 policies—transitioning from a dynamic zero-COVID approach to the eventual classification and management of COVID-19 as a Class B infectious disease—a significant resurgence in pertussis cases was noted in 2023 (7). And reported pertussis cases increased from 1,512 in June 2023 to 97,669 by May 2024, with mortality increasing dramatically from ≤3 annual deaths (2010–2022) to 24 fatalities reported January-June 2024 (8).

For infants >2 months old with macrolide-resistant pertussis, compound sulfamethoxazole (SMZco) represents an alternative treatment. However, real-world evidence of its pediatric effectiveness remains limited. This retrospective study evaluates the comparative clinical effectiveness and safety of SMZco vs. macrolides in pediatric pertussis management, providing evidence for clinical decision-making in high-resistance settings.

## 2 Patients and methods

### 2.1 Patients

We enrolled pediatric patients (aged ≥2 months) with laboratory-confirmed *Bordetella pertussis* infection via PCR testing at Ziyang People's Hospital between January and December 2024. Demographic and clinical data including gender, age, symptoms, and adverse drug reactions were collected. The therapeutic regimen, comprising either a macrolide antimicrobial agent or SMZco, was selected in accordance with the family's decision, which factored in the potential for adverse drug reactions. Participants were stratified into macrolide and SMZco (including patients who were initially treated with macrolide antimicrobial drugs but showed inadequate clinical response, and were subsequently switched to SMZco for continued therapy) treatment groups based on therapeutic regimen. The study was approved by the Ethics Committee of Ziyang People's Hospital, with written informed consent obtained from all legal guardians.

### 2.2 Inclusion and exclusion criteria

#### 2.2.1 Inclusion criteria

Diagnosis required fulfillment of both:

1. Clinical manifestations: paroxysmal spasmodic coughing with/without whooping, post-tussive vomiting, apnea episodes, or nocturnal coughing
2. Laboratory confirmation: PCR-positive for *B. pertussis (*9)

#### 2.2.2 Exclusion criteria

1. Concurrent severe infections with other pathogens or complicated pneumonia
2. Hepatic/renal dysfunction (ALT >2× ULN, Cr >1.5× ULN)
3. Documented hypersensitivity to erythromycin or SMZco

### 2.3 Treatment protocols

#### 2.3.1 Antibiotic therapy

- Macrolide group:
  ○ Erythromycin (Hainan Aomeihua Pharma; 0.15 g/sachet; NMPA approval H20090269): 30–50 mg/kg/day divided bid
  ○ Azithromycin (Liaoning Tianlong Pharma; 0.1 g/sachet; NMPA approval H20064332): 10 mg/kg/day qd
- SMZco group:
  ○ Co-trimoxazole [Xiamen Xingsha Pharma; 0.48 g/tablet (TMP 80 mg/SMX 400 mg); NMPA approval H35020249]: 48 mg SMZco/kg/day divided bid

#### 2.3.2 Supportive treatment

Expectorant, nebulization and other symptomatic treatments.

### 2.4 Outcome measures

#### 2.4.1 Primary endpoint—therapeutic effectiveness at day 14

- **Markedly effective**: Complete resolution of symptoms
- **Effective**: Symptoms of spasmodic cough, cough vomiting and nocturnal cough were relieved.
- **Ineffective**: No significant symptom improvement

#### 2.4.2 Secondary endpoints

- Duration of key symptoms (spasmodic coughing, post-tussive vomiting, nocturnal coughing)
- Hospitalization length (days)
- Incidence of adverse events (rash, gastrointestinal reactions)

### 2.5 Statistical analysis

Data were analyzed using Microsoft Office Excel 2019. Continuous variables were expressed as mean ± SD (Student's *t*-test), while categorical data were presented as counts/percentages (Chi-square/Fisher's exact test). We utilized the Cox proportional hazards regression model to compare the Nocturnal Cough Duration, Paroxysmal Cough Duration, Post-tussive Vomiting Duration and Hospitalization Length between the two patient groups, calculating the hazard ratios and their 95% confidence intervals. Statistical significance was set at *p* < 0.05 (two-tailed).

## 3 Results

### 3.1 Baseline characteristics

A total of 102 pediatric patients were enrolled in this study, with equal gender distribution (51 males, 51 females) and a mean age of 6.4 ± 2.6 years. All participants had completed pertussis vaccination. The cohort comprised 23 patients in the macrolide group (11 males, 12 females) and 79 (including 11 patients whose therapy was switched from macrolides to SMZco) in the SMZco group (40 males, 39 females).Gender analysis revealed no significant intergroup difference (*χ*^2^ = 0.056, *p* = 0.814). However, the macrolide group demonstrated significantly older age (7.9 ± 1.8 years vs. 5.9 ± 2.7 years; t = −3.337, mean difference = 2.0 years, 95% CI: 0.81–3.19, *p* = 0.001). Disease duration at presentation showed comparable results between groups (19.5 ± 9.4 days vs. 15.2 ± 9.6 days; t = −1.899, mean difference = 4.3 days, 95% CI: −0.19–8.79, *p* = 0.061).Radiographic evaluation was completed in 22 macrolide-treated patients (17 with pneumonia signs) and 76 SMZco recipients (39 with pneumonia signs), demonstrating significant intergroup disparity in pulmonary involvement (*χ*^2^ = 4.465, *p* = 0.035) (Table 1).

**Table 1:** Baseline characteristics of study participants.

Group	Gender		Age (years)	Disease duration (days)	Radiographic pneumonia (*n*)	WBC (×10^9^ /L)	Lymphocyte (%)
	Male	Female					
Macrolide	11	12	7.9 ± 1.8	19.5 ± 9.4	17	10.7 ± 3.9	39.2 ± 9.9
SMZco	40	39	5.9 ± 2.7	15.2 ± 9.6	39	10.2 ± 3.9	40.1 ± 13.6

### 3.2 Hematological parameters

Comparative analysis of hematological parameters between the two groups demonstrated no statistically significant differences. The white blood cell (WBC) count in the macrolide group (10.7 ± 3.9 × 10^9^ /L) was comparable to that in the SMZco group (10.2 ± 3.9 × 10^9^ /L; *t* = −0.541, MD = 0.5, 95% CI: −1.33–2.33, *p* = 0.589). Similarly, lymphocyte percentages showed no significant variation, with values of 39.2 ± 9.9% and 40.1 ± 13.6% in the macrolide and SMZco groups, respectively (*t* = 0.295, MD = 0.9, 95% CI: −5.15–6.91, *p* = 0.769; Table 1).

### 3.3 Treatment effectiveness

The SMZco group exhibited significantly superior therapeutic effectiveness compared to the macrolide group. In the macrolide cohort, 11 of 23 patients (47.8%) achieved clinical improvement (3 markedly effective, 8 effective), whereas 64 of 79 patients (81.0%) in the SMZco group demonstrated positive outcomes (26 markedly effective, 38 effective) (Table 2). A multiple linear regression analysis was performed to assess the impact of age, disease duration, and chest imaging findings on treatment outcomes between the two groups. The adjusted *R*^2^ was 0.01121, indicating that age and other two factors could only explain 1.12% of the results. *F* = 1.3666 and *P* = 0.2578 > 0.05, suggesting the regression model had no significant statistical significance. Analysis of the individual effect of the three factors on the treatment effectiveness revealed no statistically significant impact for any of them (*P* > 0.05 for each) (Table 3).

**Table 2:** Comparative analysis of treatment effectiveness between study groups.

Group	Total cases	Markedly effective (*n*)	Effective (*n*)	Ineffective (*n*)	Total effective cases (*n*)
Macrolide	23	3	8	12	11
SMZco	79	26	38	15	64

**Table 3:** Multiple regression analysis of treatment effectiveness between study groups.

Independent variables	Coefficient	Std. error	t	*P*	r_partial_	r_semipartial_	VIF	*R*^2^-adjusted	F-ratio	Significance level
(Constant)	1.0111							0.01121	1.3666	*P* = 0.2578
Disease Duration	−0.03289	0.02037	−1.615	0.1098	−0.1643	0.163	1.085			
Radiographic Pneumonia	−0.008456	0.09507	−0.0889	0.9293	−0.009174	0.00898	1.088			
Age	−0.001236	0.001491	−0.829	0.4093	−0.08518	0.08369	1.079			

### 3.4 Secondary clinical outcomes

SMZco treatment was associated with accelerated symptom resolution and reduced healthcare utilization. Patients receiving macrolides experienced prolonged nocturnal coughing (8.7 ± 4.5 days vs. 5.5 ± 2.0 days; *t* = −4.9, MD = 3.2, 95% CI: 1.61–4.79, *p* = 0.001; OR = 2.9412, 95% CI: 1.6947, 5.0998, *P* = 0.0001) and extended paroxysmal coughing duration (8.3 ± 2.9 days vs. 4.9 ± 1.3 days; *t* = −8.06, MD = 3.4, 95% CI: 2.56–4.24, *p* < 0.0001; OR =  6.7114, 95% CI: 3.3285, 13.5320, *P* < 0.0001). Post-tussive vomiting persisted longer in the macrolide group (1.4 ± 1.2 days vs. 0.9 ± 1.0 days; *t* = −2.0, MD = 0.5, 95% CI: 0.0077–0.99, *p* = 0.046; OR = 1.4706, 95% CI: 0.9207, 2.3515, *P* = 0.1089). Furthermore, hospitalization duration was significantly shorter for SMZco recipients (8.8 ± 2.4 days vs. 10.7 ± 2.5 days; *t* = −3.31, MD = 1.9, 95% CI: 0.76–3.04, *p* = 0.001; OR = 1.8519, 95% CI:1.1494, 2.9744, *P* = 0.0103), underscoring its clinical and economic advantages (Table 4).

**Table 4:** Comparison of symptom duration and hospitalization length between treatment groups.

Group	NCD (days)	PCD (days)	PVD (days)	HL (days)
Macrolide	8.7 ± 4.5	8.3 ± 2.9	1.4 ± 1.2	10.7 ± 2.5
SMZco	5.5 ± 2.0	4.9 ± 1.3	0.9 ± 1.0	8.8 ± 2.4

*NCD, nocturnal cough duration; PCD, paroxysmal cough duration; PVD, post-tussive vomiting duration; HL, hospitalization length.

### 3.5 Analysis of adverse events

Comparative analysis of adverse drug reactions revealed distinct safety profiles between the treatment groups. Gastrointestinal adverse effects were significantly more prevalent in the macrolide group (8/23 cases, 34.8%) compared to the SMZco group (6/79 cases, 7.6%; *χ*^2^ = 11.01, *p* = 0.0009). Conversely, cutaneous manifestations (rash) were exclusively observed in the SMZco group (12/79 cases, 15.2%), demonstrating statistically higher incidence than the macrolide group (0/23 cases; *χ*^2^ = 3.92, *p* = 0.047) (Table 5).

**Table 5:** Comparative analysis of adverse event incidence between treatment groups.

Group	Total cases (*n*)	Gastrointestinal reactions (*n*)	Cutaneous rash (*n*)
Macrolide	23	8	0
SMZco	79	6	12

## 4 Discussion

Pertussis, an acute respiratory infection caused by *Bordetella pertussis*, is clinically characterized by paroxysmal spasmodic coughing with inspiratory whooping. While global pertussis incidence declined significantly following the 1974 introduction of diphtheria-tetanus-pertussis (DTP) vaccine and China's 1978 national immunization program. In China, a marked upward trend in reported pertussis cases was observed between 2016 and 2019, with case numbers rising from 5,584 in 2016 to over 30,000 by 2019. After a significant decline during the COVID-19 containment period, pertussis incidence rebounded markedly following the policy shift, with a sharp rise in cases observed in 2023 (7).

Macrolides have traditionally served as first-line therapy, but emerging resistance poses growing challenges (10). This resistance evolution may correlate with increasing ptxP3 strain prevalence (6). Additional mechanisms involve 23S rRNA A2047G mutations, universally detected in resistant isolates but absent in susceptible strains (11). The molecular basis of macrolide resistance warrants further investigation.

Compound sulfamethoxazole (SMZco) exerts bactericidal effects through dual inhibition of dihydropteroate synthase (competing with PABA) and dihydrofolate reductase, disrupting folate metabolism and consequently nucleic acid/protein synthesis. This mechanism circumvents typical macrolide resistance pathways. Our findings demonstrate superior effectiveness of SMZco (81.0% response rate) vs. macrolides (47.8%, *p* < 0.05), with significant reductions in hospitalization duration and symptom persistence (all *p* < 0.05), aligning with prior research (12). We performed a multiple linear regression to evaluate the influence of age, disease duration, and chest imaging findings on the therapeutic effectiveness in both groups, indicating the regression model had no significant statistical significance. Radiographic evaluation was completed in 22 macrolide-treated patients (17 with pneumonia signs) and 76 SMZco recipients (39 with pneumonia signs), demonstrating significant intergroup disparity in pulmonary involvement (*χ*^2^ = 4.465, *p* = 0.035). However, this discrepancy did not significantly influence the final therapeutic effectiveness. We postulate that this may be attributed to the concurrent intravenous administration of other antibiotics, such as third-generation cephalosporins or penicillins, alongside macrolide antibiotics and SMZco, to treat potential co-infections caused by other bacteria.

The safety analysis revealed divergent adverse effect profiles: gastrointestinal reactions predominated with macrolides (8/23 cases, 34.8%) vs. SMZco (6/79, 7.6%; *p* < 0.05), while cutaneous rash exclusively occurred with SMZco (12/79, 15.2% vs. 0/23; *p* < 0.05).

The rising prevalence of macrolide-resistant *B. pertussis* strains presents a significant public health challenge, particularly in regions with high vaccination coverage yet persistent pertussis transmission. Our findings highlight several critical considerations:

1. **Antibiotic Stewardship**—The high effectiveness of SMZco against resistant strains supports its inclusion in empirical treatment guidelines, particularly in areas with documented macrolide resistance. This could help mitigate treatment failures and reduce secondary transmission.
2. **The Timing of Administration—**In our study, the disease duration was 19.5 ± 9.4 days in the macrolide group and 15.2 ± 9.6 days in the SMZco group, suggesting that even when administered after 2 weeks of illness onset, the treatment can still alter the disease outcome.
3. **Vaccination Policy Reassessment**—The emergence of MR-MT28 and other resistant clones capable of infecting vaccinated individuals suggests potential gaps in long-term immunity. Booster vaccination strategies, particularly for older children and adolescents, may be necessary to curb transmission.
4. **Surveillance and Resistance Monitoring**—Enhanced genomic surveillance of circulating *B. pertussis* strains is essential to track resistance patterns and guide regional treatment recommendations. Early detection of novel resistance mechanisms can inform timely updates to clinical guidelines.
5. **Economic Burden Reduction**—The shorter hospitalization and symptom duration observed with SMZco suggest potential cost savings for healthcare systems, particularly in outbreak settings where resource allocation is critical.
6. **Global Health Considerations**—Given the international spread of resistant strains, coordinated efforts between public health agencies are needed to establish standardized resistance reporting and optimize treatment protocols across borders.

Several limitations should be acknowledged in this study. First, the retrospective design may introduce potential selection bias due to non-randomized treatment allocation. Second, the assessment of symptom duration relied on medical records, which may be subject to documentation inaccuracies. Third, the relatively small sample size, particularly in the macrolide group (*n* = 23), along with statistically significant baseline differences in age and radiographic findings between groups, may impact the validity of our results. These methodological constraints highlight the need for larger-scale prospective studies with rigorous randomization and standardized outcome measures to confirm these preliminary findings. Future research should aim to enroll more balanced cohorts while incorporating molecular characterization of resistant strains to further validate the clinical utility of TMP-SMX in macrolide-resistant pertussis.

## Conclusion

Amid global pertussis resurgence and escalating macrolide resistance, SMZco demonstrates significantly enhanced therapeutic effectiveness, accelerated symptom resolution, and reduced hospitalization. These findings position SMZco as an effective and safe alternative, particularly in regions with high macrolide resistance.

## Data Availability

The original contributions presented in the study are included in the article/Supplementary Material, further inquiries can be directed to the corresponding author.

## References

[1] Vásconez NS Jaramillo K Zabala A Villacís JE. Bordetella pertussis, a reemerging pathogen in pediatric respiratory infections. A study in Quito, Ecuador. Rev Argent Microbiol. (2021) 53:27–33. 10.1016/j.ram.2020.07.001 33243445

[2] Liu TC Zhang J Liu SQ Yin AT Ruan SM. Evaluation of immunisation strategies for pertussis vaccines in Jinan, China—an interrupted time-series study. Epidemiol Infect. (2020) 148:e26. 10.1017/S0950268820000102 32046804 PMC7026899

[3] Fu P Zhou J Yang C Nijiati Y Zhou L Yan G Molecular evolution and increasing macrolide resistance of Bordetella pertussis, Shanghai, China, 2016–2022. Emerg Infect Dis. (2023) 30(1):29–38. 10.3201/eid3001.221588 38146984 PMC10756392

[4] Li L Deng J Ma X Zhou K Meng Q Yuan L High prevalence of macrolide-resistant Bordetella pertussis and ptxP1 genotype, mainland China, 2014–2016. Emerg Infect Dis. (2019) 25:2205–14. 10.3201/eid2512.181836 31742507 PMC6874251

[5] Ivaska L Barkoff A Mertsola J He Q. Macrolide resistance in Bordetella pertussis: current situation and future challenges. Antibiotics. (2022) 11:1570. 10.3390/antibiotics11111570 36358225 PMC9686491

[6] Cai J Chen M Liu Q Luo J Yuan L Chen Y Domination of an emerging erythromycin-resistant ptxP3 Bordetella pertussis clone in Shanghai, China. Int J Antimicrob Agents. (2023) 62:106835. 10.1016/j.ijantimicag.2023.106835 37127126

[7] Mengyang G Yahong H Qinghong M Wei S Kaihu Y. Resurgence and atypical patterns of pertussis in China. J Infection. (2024) 88:106140. 10.1016/j.jinf.2024.106140 38513738

[8] Yahong H Mengyang G Meng Q Yu D Kaihu Y. Rising pertussis cases and deaths in China: current trends and clinical solutions. Emerg Microbes Infec. (2024) 13(1):2389086. 10.1080/22221751.2024.2389086 39101270 PMC11340210

[9] [Guidelines for diagnosis and management and prevention of pertussis of China (2024 edition)]. Zhonghua Yi Xue Za Zhi. (2024) 104:1258–79. 10.3760/cma.j.cn112137-20240124-00179 38637166

[10] Mi YM Hua CZ Fang C Liu JJ Xie YP Lin LN Effect of macrolides and *β*-lactams on clearance of Bordetella pertussis in the nasopharynx in children with whooping cough. Pediatr Infect Dis J. (2021) 40:87–90. 10.1097/INF.0000000000002911 33021592

[11] Zhang JS Zhang DQ Lin C Chang L Ma CF Chen BZ Comparison of macrolide resistance, molecular characteristics and MAST types of Bordetella pertussis collected from Xi'an and Shanghai. J.Xi'an Jiaotong Univ (Medical Sciences). (2022) 43:691–6.

[12] Wang Y He LT Chen LY Pan JH Zhang L. Effect observation of compound sulfamethoxazole in the treatment of pertussis in children. Chin J Gen Pract. (2024) 22:614–7. 10.16766/j.cnki.issn.1674-4152.003463

